# Optimised knowledge distillation for efficient social media emotion recognition using DistilBERT and ALBERT

**DOI:** 10.1038/s41598-025-16001-9

**Published:** 2025-08-17

**Authors:** Muhammad Hussain, Caikou Chen, Muzammil Hussain, Muhammad Anwar, Mohammed Abaker, Abdelzahir Abdelmaboud, Iqra Yamin

**Affiliations:** 1 College of Information and Artificial Intelligence, Yangzhou University, Yangzhou, 225000 People’s Republic of China; 2 Department of Software Engineering, Faculty of Information Technology, Al-Ahliyya Amman University, Amman, 19328 Jordan; 3 Division of Science and Technology, Department of Information Sciences, University of Education, Lahore, 54000 Pakistan; 4 Institute of Visual Informatics, Universiti Kebangsaan Malaysia, Bangi, Malaysia; 5 Applied College, King Khalid University, Muhayil, 61913 Saudi Arabia; 6 Humanities Research Center, Sultan Qaboos University, Muscat, Oman

**Keywords:** Emotion recognition, Knowledge distillation, Transformer compression, Efficient NLP, Social media emotions, DistilBERT, ALBERT, Psychology, Mathematics and computing

## Abstract

Accurate emotion recognition in social media text is critical for applications such as sentiment analysis, mental health monitoring, and human-computer interaction. However, existing approaches face challenges like computational complexity and class imbalance, limiting their deployment in resource-constrained environments. While transformer-based models achieve state-of-the-art performance, their size and latency hinder real-time applications. To address these issues, we propose a novel knowledge distillation framework that transfers knowledge from a fine-tuned BERT-base teacher model to lightweight DistilBERT and ALBERT student models, optimised for efficient emotion recognition. Our approach integrates a hybrid loss function combining focal loss and Kullback-Leibler (KL) divergence to enhance minority class recognition, attention-head alignment for effective contextual knowledge transfer, and semantic-preserving data augmentation to mitigate class imbalance. Experiments on two datasets, Twitter Emotions 416 K samples, six classes, and Social Media Emotion 75 K samples, five classes, show that our distilled models achieve near-teacher performance 97.35% and 73.86% accuracy, respectively. with only a < 1% and < 6% accuracy drop, while reducing model size by 40% and inference latency by 3.2×. Notably, our method significantly improves F1-scores for minority classes. Our work sets a new state-of-the-art in efficient emotion recognition, enabling practical deployment in edge computing and mobile applications.

## Introduction

Emotions are fundamental to human communication, influencing decision-making, behaviour, and interpersonal relationships. In the digital age, where online interactions dominate over physical interaction, the ability to accurately recognise emotions in text has become a critical challenge, with broad applications across various domains such as customer service, healthcare, and social media analysis^1–3^. Text emotion recognition is crucial for enhancing the responsiveness of chatbots, improving healthcare communication, and refining sentiment analysis on social media, which enhances customer satisfaction and better user experiences^4,5^. However, significant challenges are present in emotion recognition. The expression of conversational emotions can be subtle, dynamic, and influenced by context, including speaker characteristics and the surrounding conversational environment^6^. This dynamic nature of emotional expression makes it difficult to distinguish between similar emotional expressions and to classify them accurately. Excitement expressions of emotional context and happiness emotional context may be vague in certain contexts, leading to model misclassification of these expressions^7^. Furthermore, the expression of emotion contagion and dynamic changes in public opinion identify the public mood before and after natural disasters^8^, identifying the preference during the election voting campaign public opinions^9^ and prediction on stock market^10^ topics and implicit knowledge shared between participants in conversations can alter the meaning and emotional tone of context, complicating the task of emotion recognition. However, the dynamic, noisy, and informal nature of social media data presents significant challenges for emotion recognition, especially in terms of scalability and accuracy. Traditional approaches to emotion recognition rely heavily on large language and complex models, such as BERT^11^, RoBERTa^12^, and GPT^13^, which provide high accuracy but suffer from significant computational costs, which makes them challenging to deploy such models in resource-constrained environments where fast inference and reduced model size are essential. Consequently, there is a growing need for efficient models that can provide comparable performance while being more computationally efficient.

Despite significant advancements in natural language processing (NLP), accurately recognising emotions in social media text remains a complex challenge. A major issue is the trade-off between model accuracy and efficiency. While the BERT transformer-based large language model and its variants, transformer-based large language models, achieve state-of-the-art performance on NLP tasks, emotion recognition, and sentiment analysis, these models are computationally expensive, require more substantial resources, and are often prohibitively large for training and inference. Therefore, an efficient and scalable approach is needed to make the emotion recognition model feasible for real-time applications in resource-constrained environments, such as mobile and edge devices. To mitigate these challenges, we introduce a novel solution that combines the strengths of knowledge distillation^14^ and lightweight transformer models, DistilBERT and ALBERT^15,16^, an efficient and scalable context emotion recognition on social media platforms, which required limited resources. Knowledge distillation (KD) offers a promising solution by transferring the knowledge from a large teacher model to a smaller student model, thus improving efficiency without losing accuracy and performance. We employ BERT-base as the teacher model and DistilBERT and ALBERT as separate student models for the knowledge distillation process. We trained a KD model on two popular public datasets available on the Kaggle public repository, the twitter emotion dataset and the social media emotion dataset. Our approach does not need the overhead of computational resources while maintaining high performance in recognising nuanced emotional expressions without compromising performance.

Our contributions are as follows:

- Scalable Emotion Recognition via Lightweight Transformers: We introduce a scalable emotion recognition framework that employs computationally efficient transformer-based architectures (DistilBERT and ALBERT). These models are optimised for reduced resource consumption while preserving high accuracy, enabling deployment in resource-constrained environments without sacrificing performance.
- Knowledge Distillation for Model Efficiency: To bridge the performance gap between large and lightweight models, we apply knowledge distillation (KD) to transfer learned representations from BERT-base to distilled variants (DistilBERT and ALBERT). This approach achieves competitive accuracy with significantly lower computational overhead, enhancing practicality for real-world applications.
- Efficient Emotion Recognition in Social Media: Our proposed framework is optimised for the heterogeneous and rapidly evolving emotional expressions dominant in social media, leveraging adaptive learning mechanisms to ensure high robustness and detection accuracy across diverse linguistic and contextual variations.
- Class Imbalance Mitigation via Advanced Learning Strategies: We counteract the inherent class imbalance in emotion datasets by integrating focal loss, enhancing model sensitivity toward underrepresented emotional classes while maintaining overall classification performance.
- Comprehensive Evaluation on Real-World Social Media Data: We rigorously evaluate our framework on diverse, real-world social media datasets collected from twitter and social media datasets, carefully curated to reflect imbalance in emotional expressions.

Our research significantly advances real-time emotion recognition for social media by introducing a computationally efficient framework deployable on resource-constrained mobile and edge devices. The implications of this work span multiple domains: for sentiment analysis, it enhances detection of nuanced emotions like sarcasm and mixed feelings in short-text social media posts; in public opinion monitoring, it facilitates real-time tracking of emotional trends at scale; and for recommendation systems, it enables emotion-aware content personalisation^17,18^.These advancements open new possibilities for deploying sophisticated emotion recognition in real-world scenarios where both efficiency and accuracy are paramount.

## Literature review

Emotion recognition in social media text, particularly from twitter, has emerged as a critical task in natural language processing (NLP). The capability to accurately recognise and classify emotional states, such as joy, anger, sadness, or fear, from user-generated content offers significant value across multiple domains. In business and marketing, it enables real-time customer sentiment analysis and brand perception tracking. For public health applications, it facilitates large-scale mental health monitoring by identifying emotional distress signals in user posts. In finance, emotion-driven analytics can enhance stock market prediction models by capturing investor sentiment. Political analysts leverage these techniques to measure public opinion and emerging trends. The challenge lies in developing robust models that can handle the noisy, informal nature of social media text while accounting for linguistic nuances like sarcasm, irony, and culturally context-dependent expressions^19^. Traditional methods in emotion recognition often relied on techniques that have undergone significant paradigm shifts. Early approaches predominantly relied on manual feature engineering, lexicon-based sentiment scores, n-gram patterns combined with conventional machine learning classifiers such as SVMS or Random Forests. These traditional methods faced inherent limitations in capturing contextual nuances and handling the linguistic complexity of informal social media text^20,21^. The field has since been transformed by deep learning, particularly through pre-trained language models like BERT and GPT, which have established new state-of-the-art benchmarks^22^. These transformer-based architectures demonstrate superior capability in learning hierarchical textual representations, enabling more accurate detection of subtle emotional cues, cultural references, and pragmatic elements like irony or humour that are prevalent in social media discourse.

Early emotion recognition efforts relied on feature-based methods, extracting emotion-relevant features like sentiment lexicons and handcrafted word embeddings to train classifiers such as Support Vector Machines SVMs and Random Forests^23^. While effective for simple tasks, these approaches struggled to capture the contextual nuances of emotions in text. The advent of deep learning introduced recurrent neural networks RNNs and long short-term memory networks LSTMs, which improved performance by modeling sequential data^24^. Convolutional neural networks CNNs for sentence classification have been used to achieve strong results on sentiment analysis tasks, though less effective for fine-grained emotion recognition^25^.

Despite their advancements, RNN-based models face challenges with long-range dependencies and computational inefficiencies. The growth of transformer-based models^26^ addressed these limitations through self-attention mechanisms to capture dependencies across entire input sequences, regardless of their distance. BERT is a transformer-based model pre-trained on large text corpora and fine-tuned on downstream tasks such as text classification, sentiment analysis, customer reviews, question answering, named entity recognition, and emotion recognition^27,28^. The popularity of BERT in natural language processing tasks has prompted the development of specialised variants that address its limitations through two primary approaches: performance optimisation and computational efficiency. For maximal accuracy, models like RoBERTa are rigorously optimised through dynamic masking and extended training cycles. At the same time, DeBERTa^29^ advances architectural foundations via disentangled attention mechanisms, both achieving state-of-the-art results in complex tasks like emotion recognition but maintaining substantial computational demands. Conversely, efficiency-focused variants employed innovative parameter reduction techniques such as ALBERT^16^, which dramatically decreased memory requirements through cross-layer parameter sharing and factorised embeddings, whereas DistilBERT^30^ and TinyBERT^31^ which utilised knowledge distillation, have been developed to improve performance, reduce model size, and increase training efficiency. BERT-based pre-trained models have consistently demonstrated superior performance over traditional deep learning architectures, such as Recurrent Neural Networks (RNNs) and Long Short-Term Memory (LSTM) networks, across a variety of NLP tasks. These models leverage bidirectional context through transformer-based architecture, which enhances their ability to capture intricate dependencies within text, outperforming earlier models that rely on sequential processing. As a result, BERT and its variants have become the state-of-the-art approach in many NLP domains, offering significant improvements in both accuracy and computational efficiency. Recent studies have also explored multi-modal approaches that combine textual data with other modalities, such as images and videos, to enhance emotion classification performance further^32,33^. RMER-DT^34^ and CIME^35^ have demonstrated how integrating transformer-based encoders with diffusion or contextual interaction mechanisms can significantly enhance multimodal emotion recognition by capturing deeper conversational structure and semantic dependencies. While our work focuses on unimodal text, these approaches underscore the importance of context modeling, which remains crucial in social media emotion detection. Additionally^36^, have explored efficient and contextualised architectures in speech emotion recognition, including CNN-Transformer fusion while contextual Transformer-GRU models^37^, and hierarchical attention for dyadic conversations^38^. Although focused on speech, their hybrid designs reflect a shared motivation integrating context-awareness with architectural efficiency principles, which are also central to our lightweight text-based approach. However, despite the impressive success of these models, their substantial computational demands pose a significant challenge for real-time applications, particularly in resource-constrained environments such as mobile devices or edge computing.

Knowledge distillation (KD) is a technique where a smaller, more efficient student model is trained to mimic the behaviour of a larger, more accurate teacher model^39^. The primary advantage of KD lies in its ability to enable the deployment of high-performance models that are smaller in size and more efficient in terms of memory consumption and inference speed. The KD approach has been successfully applied across various domains, including computer vision and NLP, where it has been used to distill the knowledge of large transformer models like BERT into smaller models such as DistilBERT and TinyBERT^30,31^. In NLP, knowledge distillation has become an essential tool for making state-of-the-art transformer models more accessible for deployment in practical applications. A generalised framework for knowledge generation^40^ and distillation proposed, aligning feature distributions between teacher and student models and underscoring the broader applicability of KD in constrained deployment environments. The distillation principles support the goals of the text-based approach.

Although KD has been shown to improve the efficiency of large language models significantly, its specific application in emotion detection on social media remains relatively underexplored. Most prior research on KD in NLP has focused on tasks such as question answering, text classification, and machine translation, where the goal has been to reduce model size and increase inference speed without compromising accuracy^41^. However, the potential of KD to optimise models for real-time emotion recognition, particularly on platforms like social media, has not been fully realised. Contrastive-based techniques have recently been used to improve representation quality in multimodal emotion recognition^42^. Although their methods focus on filtering negative and positive information from video and speech inputs, the underlying contrastive learning strategy uses contrastive loss to improve discriminative learning in resource-constrained text emotion models.

Our paper aims to demonstrate that a distilled model can achieve comparable accuracy to larger models while being substantially more efficient, making it more suitable for real-time applications that require rapid processing of large volumes of user-generated content.

In emotion recognition, both classification accuracy and inference speed are crucial. Given the fast-paced nature of social media and the volume of content generated, achieving the right balance between model performance and efficiency is paramount. To address this challenge, our research explores the distillation of BERT, a high-performance pre-trained transformer model, into more compact and faster alternatives such as ALBERT and DistilBERT. These distilled models retain the core capabilities of the original BERT model but are significantly more efficient in terms of memory usage and inference speed, making them better suited for real-time emotion detection applications^31,41^.

By leveraging knowledge distillation, the trade-off achieved through this distillation process makes these models more viable for deployment in resource-constrained environments, such as mobile devices or edge computing platforms, where real-time emotion detection is critical. Our findings show that distilling BERT into smaller models not only reduces computational requirements but also preserves the emotional understanding capabilities necessary for accurate emotion classification on social media platforms^14,43^.

## Datasets

**Fig. 1:**
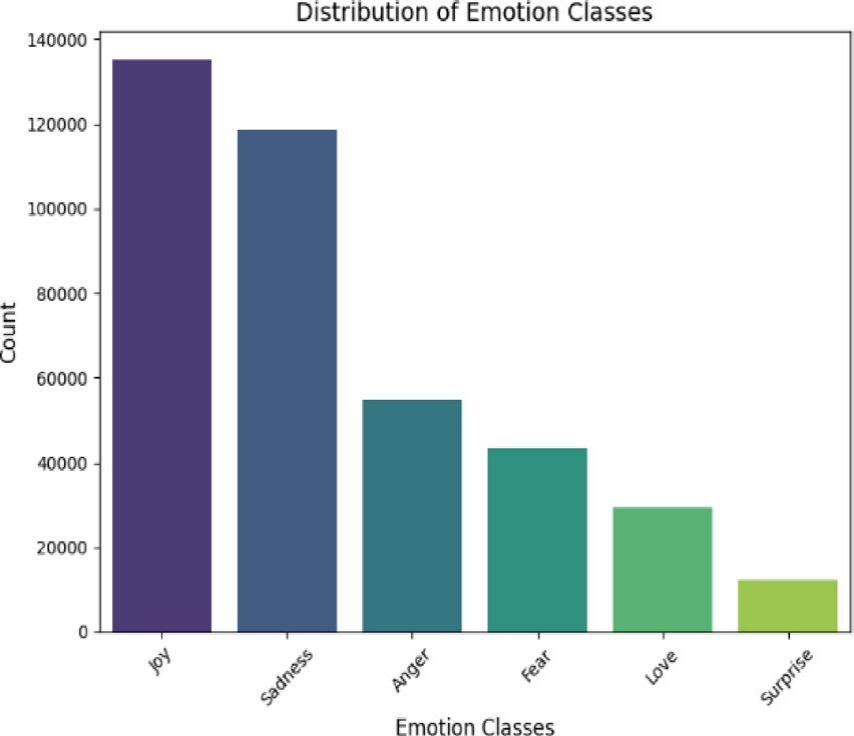
Twitter’s emotion classes.

**Fig. 2:**
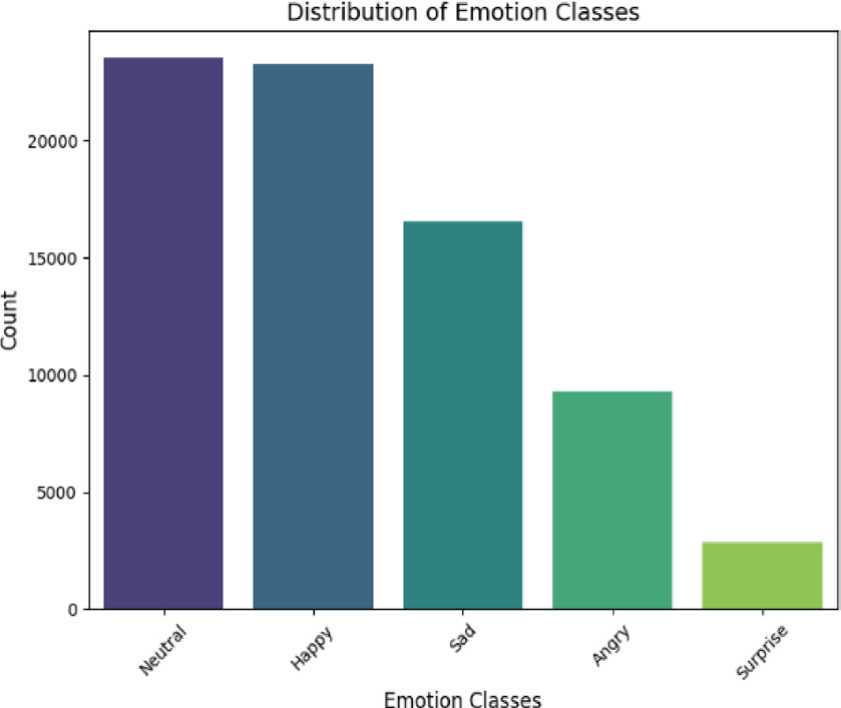
Twitter word count per emotion classes.

For our experiments, we have used two publicly available emotion classification datasets sourced from Kaggle, a prominent platform for open-source datasets. The first dataset, Twitter Emotions, consists of 416,809 tweets, each labelled with one of six emotion categories: Joy, Sadness, Anger, Fear, Love, and Surprise. The dataset exhibits a notable class imbalance, with certain emotions (e.g., Joy) appearing more frequently than others (e.g., Surprise). Figure 1 illustrates the distribution of emotion classes, while Fig. 2 provides an analysis of word count per emotion, offering insights into linguistic patterns across different emotional expressions. This dataset is particularly valuable for studying emotion recognition in short, informal social media text.

**Fig. 3:**
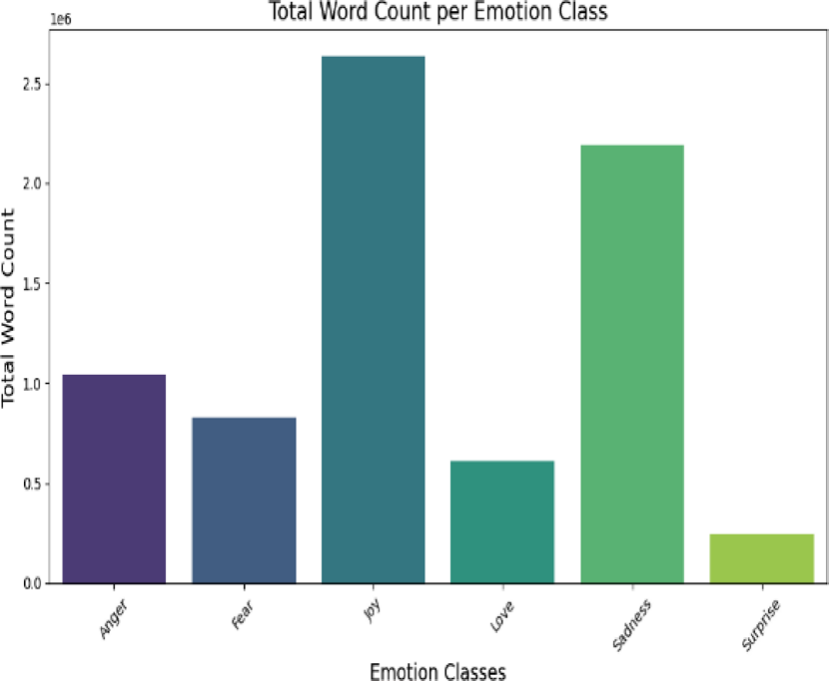
Social media emotion classes.

**Fig. 4:**
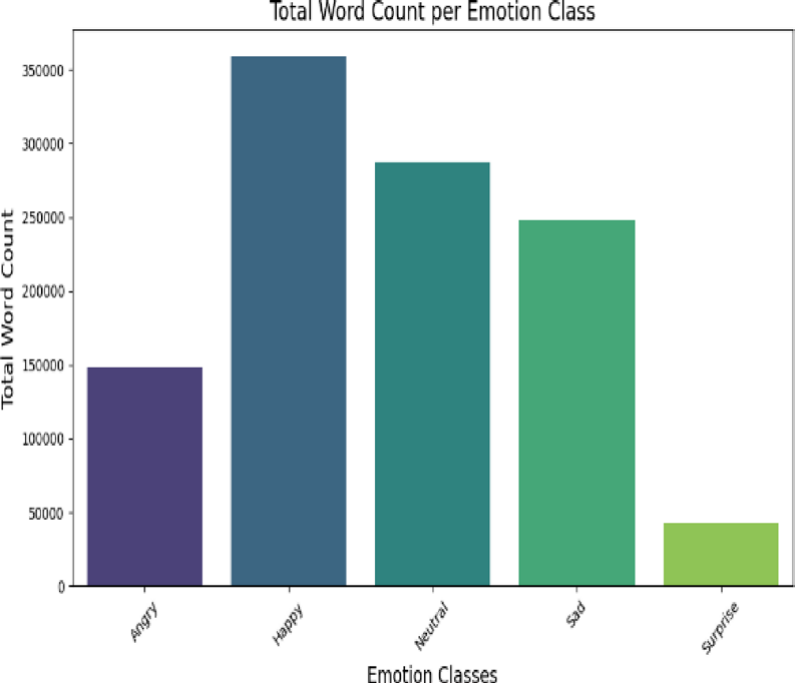
Social media word count per emotion classes.

The second dataset, Social Media Emotion, contains 75,847 text samples labelled into five emotion classes: Neutral, Happy, Sad, Anger, and Surprise. Unlike the Twitter dataset, this corpus includes a Neutral category, allowing for a more nuanced analysis of emotional versus non-emotional content. The class distribution, shown in Fig. 3, also reflects imbalance, though with different skew patterns compared to the Twitter dataset. While Fig. 4 provides an analysis of word count per emotion, both datasets are widely used in sentiment and emotion analysis research, ensuring that our experiments are grounded in established benchmarks. Their varying characteristics, Twitter’s short, noisy text versus Social Media Emotion’s broader context, enable a robust evaluation of our model’s generalizability across different types of emotional expression.

### Data preprocessing

Data preprocessing is a crucial step in preparing raw social media text for emotion recognition tasks. Our comprehensive preprocessing pipeline ensures high-quality input for the learning models while addressing the unique challenges of informal text. We fixed the random seed to 42 to guarantee consistent dataset shuffling. We used stratified splitting to preserve class distributions across the training, validation, and testing splits, with 70% allocated for training, 15% for validation, and 15% for testing for both datasets.

#### Data cleaning

The data cleaning process involved several key steps to ensure dataset quality. Null Value Handling: We removed all instances containing null values to maintain dataset integrity^44^. Text Normalisation: Punctuation removal to reduce noise, and Emoji elimination to focus on textual content. Stopword removal using NLTK’s standard stopword list to emphasise meaningful terms. Case Normalisation: Converted all text to lowercase for consistency. These cleaning steps significantly improved in our datasets while preserving the essential emotional content^45,46^.

#### Text tokenisation

For effective model processing, we implemented a specialised tokenisation approach: Utilised the BERT tokeniser bert-base-uncased^47^ for subword tokenisation established a maximum sequence length of 128 tokens applied dynamic padding truncation to maintain uniform input dimensions generated two key outputs: Token IDs Xids: Numerical representations of the text Attention masks Xmask: Binary indicators of token positions this tokenisation strategy effectively transformed raw text into a format suitable for transformer-based models while preserving linguistic and emotional nuances.

## Proposed methodology

**Fig. 5:**
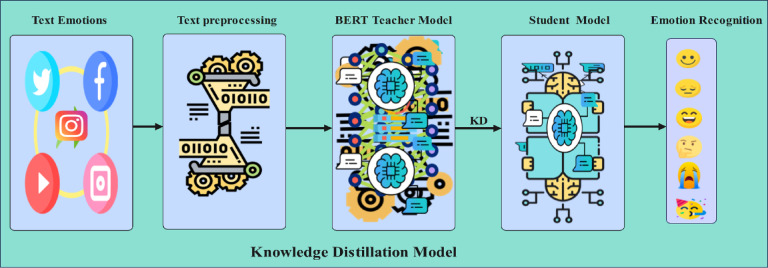
Knowledge distillation process.

Our knowledge distillation framework leverages BERT-base as the teacher model and DistilBERT/ALBERT as student models to achieve an optimal balance between accuracy and computational efficiency for deployment on resource-constrained edge devices. Figure 5 illustrates the complete framework architecture, including knowledge distillation, training, and evaluation processes.

### Model architecture

#### Teacher model BERT-base

Parameters: 110 M Architecture: 12 transformer layers, 768 hidden units. Pretrained on BooksCorpus and English Wikipedia. Fine-tuning: A dense layer with softmax activation was added for multi-class classification^27^ .

#### **Student models: DistilBERT**

Parameters: 66 M 40% reduction Architecture: 6 transformer layers Advantages: Maintains 97% of BERT’s performance with 60% faster inference^30^.

#### ALBERT

Parameters: 12 M 89% reduction Architecture: Parameter sharing and factorised embeddings. Advantages: Minimal memory footprint, ideal for mobile devices^16^. Both student models employ identical classification heads, dense + softmax, for task consistency.

### Knowledge distillation process

#### Two-stage knowledge distillation with focal loss regularisation

Let $\:(\mathcal{X},\mathcal{Y})$ be the input and label space, respectively, with $\:\mathcal{Y}=\{1,\dots\:,C\}$ representing $\:C$ emotion classes. Let $\:{\mathcal{D}}_{\text{train}}=\left\{\right({x}_{i},{y}_{i}){\}}_{i=1}^{N}$ and $\:{\mathcal{D}}_{\text{val}}$ be the training and validation sets, respectively, from the data distribution $\:\mathcal{P}(x,y)$. We define a two-stage distillation-based training framework for deep emotion recognition, where:

- $\:{T}_{\theta\:}:X\to\:{{\Delta\:}}^{C-1}$ is the teacher network with parameters $\:\theta\:$, mapping inputs to the probability simplex $\:{{\Delta\:}}^{C-1}\subset\:{\mathbb{R}}^{C}$.
- $\:{S}_{\phi\:}:X\to\:{{\Delta\:}}^{C-1}$ is the student network with parameters $\:\phi\:$, similarly, mapping inputs to $\:{{\Delta\:}}^{C-1}$.
- $\:{{\Delta\:}}^{C-1}$ denotes the probability simplex, the set of probability distributions over $\:C$ classes.
- $\:p={T}_{\theta\:}\left(x\right)$ and $\:q={S}_{\phi\:}\left(x\right)$ are the predictive probability distributions over classes for input $\:x\in\:X$.

#### Stage 1: teacher model fine-tuning

The teacher network $\:{\mathcal{T}}_{\theta\:}$ is optimised by minimising the expected focal loss $\:{\mathcal{L}}_{\text{FL}}\left(\theta\:\right)$, defined as:

1 $$\:\begin{array}{c}{\mathcal{L}}_{\text{FL}}\left(\theta\:\right)={\mathbb{E}}_{(x,y)\sim\:\mathcal{P}}\left[-{\alpha\:}_{y}(1-{p}_{y}{)}^{\gamma\:}\text{l}\text{o}\text{g}({p}_{y})\right],\:\gamma\:>0,\:{\alpha\:}_{y}\in\:\left(\text{0,1}\right)\end{array}$$

where:

- $\:{p}_{y}=\left[{\mathcal{T}}_{\theta\:}\right(x){]}_{y}=\frac{{e}^{{z}_{y}}}{\sum\:_{c=1}^{C}\:\:{e}^{{z}_{c}}}$, with $\:z=\text{logits}\left({\mathcal{T}}_{\theta\:}\right(x\left)\right)$ $\:\in\:{\mathbb{R}}^{C}$, the logits for class $\:y\in\:\text{Y}$.
- $\:{\alpha\:}_{y}\in\:\left(\text{0,1}\right)$ is a class-dependent weighting factor that mitigates class imbalance by emphasising minority classes.
- $\:\gamma\:>0$ is a focusing parameter that reduces the loss contribution of well-classified examples.
- $\:{\mathbb{E}}_{(x,y)\sim\:P}$ denotes the expectation over the data distribution $\:P(x,y)$.

The optimisation objective for the teacher is:

- The optimisation objective becomes:
  2 $$~~~~~~~~~~~~~~~~~~~~~~~~~~~~~~~~~~~~~~~~~~~~~~~~~~~~~~~~~~~~~~~~~~~~~~~~~~~~~~~~~~~~~~~~~~~~~~~~~~~~~~~~~~~~~~~~~~~~~~~~~~\begin{array}{c}                                         {\theta}^{\text{*}}=arg~\underset{\theta}{\text{m}\text{i}\text{n}}{[\mathcal{L}}_{\text{FL}}(\theta)+\lambda R(\theta)]                    \end{array}~~~~~~~~~~~~~~~~~~~~~~~~~~~~~~~~~~~~~~~~~~~~~~~~~~~~~~~~~~~~~~~~~~~~~~~~~~~~~~~~~~~~~~~~~~~~~~~~~~~~~~~~~~~~~~~~~~~~~~~~~~~~~~~~~~~$$

where:

- $\:R\left(\theta\:\right)=\Vert\:\theta\:{\Vert\:}_{2}^{2}$ is the $\:{\mathcal{l}}_{2}$-norm regularisation term to prevent overfitting by penalising large parameter values.
- $\:\lambda\:>0$ is the regularisation strength hyperparameter.
- Training employs gradient clipping, $\:‖{\nabla\:}_{\theta\:}{L}_{\text{FL}}‖\le\:G$, and early stopping based on performance on $\:{D}_{\text{val}}$.

#### Stage 2: student model distillation

The student model $\:{\mathcal{S}}_{\phi\:}$ is trained using a temperature-scaled KL divergence loss combined with focal loss. Define soft distributions:

3 $$\:\begin{array}{c}\widetilde{p}=\sigma\:\left(\frac{{z}^{\mathcal{T}}}{T}\right),\:\widetilde{q}=\sigma\:\left(\frac{{z}^{\mathcal{S}}}{T}\right)\end{array}$$

where:

- $\:{z}^{T}=\text{logits}\left({T}_{\theta\:}\right(x\left)\right)\in\:{\mathbb{R}}^{C}$ and $\:{z}^{S}=\text{logits}\left({S}_{\phi\:}\right(x\left)\right)\in\:{\mathbb{R}}^{C}$ are the teacher and student logits, respectively.
- $\:\sigma\:(\cdot\:)$ is the softmax function, $\:\sigma\:(z{)}_{i}=\frac{{e}^{{z}_{i}}}{\sum\:_{c=1}^{C}\:\:{e}^{{z}_{c}}}$.
- $\:T>0$ is the temperature hyperparameter controlling the softness of the distributions.

The distillation loss is:

4 $$\:\begin{array}{c}{L}_{\text{KD}}\left(\phi\:\right)={T}^{2}\cdot\:{\mathbb{E}}_{x\sim\:{P}_{X}}\left[\text{KL}(\widetilde{p}\Vert\:\widetilde{q})\right]\end{array}$$

where:

- $\:\text{KL}(\widetilde{p}\Vert\:\widetilde{q})=\sum\:_{c=1}^{C}\:{\widetilde{p}}_{c}\text{l}\text{o}\text{g}\left(\frac{{\widetilde{p}}_{c}}{{\widetilde{q}}_{c}}\right)$ is the KL divergence between the softened teacher and student distributions.
- $\:{T}^{2}$ scales the loss to account for the temperature’s effect on the softmax gradients.
- $\:{\mathbb{E}}_{x\sim\:{P}_{X}}$ denotes the expectation over the marginal input distribution $\:{P}_{X}$.

The total student loss is a convex combination of the distillation and focal losses:

5 $$\:\begin{array}{c}{L}_{\text{Total}}\left(\phi\:\right)=\alpha\:\cdot\:{L}_{\text{KD}}\left(\phi\:\right)+(1-\alpha\:)\cdot\:{L}_{\text{FL}}\left(\phi\:\right)\end{array}$$

where:

- $\:{L}_{\text{FL}}\left(\phi\:\right)={\mathbb{E}}_{(x,y)\sim\:P}\left[-{\alpha\:}_{y}(1-{q}_{y}{)}^{\gamma\:}\text{l}\text{o}\text{g}({q}_{y})\right]$, with $\:{q}_{y}=\left[{S}_{\phi\:}\right(x){]}_{y}=\frac{{e}^{{z}_{y}^{S}}}{\sum\:_{c=1}^{C}\:\:{e}^{{z}_{c}^{S}}}$, defined analogously to Eq. (1) using student predictions.
- $\:\alpha\:\in\:\left[\text{0,1}\right]$ is a hyperparameter balancing the teacher’s soft predictions and the ground-truth labels.
- $\:T=1.5$, so $\:{T}^{2}=2.25$, is the softening factor.

The student parameters are optimised as:

6 $$\:\begin{array}{c}{\phi\:}^{\text{*}}=arg~\underset{\phi\:}{\text{m}\text{i}\text{n}}\:\left[{L}_{\text{Total}}\left(\phi\:\right)+{\lambda\:}^{{\prime\:}}R\left(\phi\:\right)\right]\end{array}$$

where:

- $\:R\left(\phi\:\right)=\Vert\:\phi\:{\Vert\:}_{2}^{2}$ is the $\:{\mathcal{l}}_{2}$-norm regularisation term to prevent overfitting of the student model.
- $\:{\lambda\:}^{{\prime\:}}>0$ is the regularisation strength hyperparameter for the student.

Optimisation includes optional gradient clipping and early stopping based on $\:{D}_{\text{val}}$.

#### Model evaluation

Given the final trained model $\:{f}_{\widehat{\theta}}$ or $\:{f}_{\widehat{\phi}}$, performance on a test distribution $\:{\mathcal{P}}_{\text{test}}$ is measured via standard classification metrics computed as:

- Accuracy: $$\:\mathbb{P}(\widehat{y}=y)$$,
- Precision, Recall, and F1-score: weighted macro averages over $\:C$ classes.

Each student is trained independently for 20 epochs (learning rate = 1e-5), using attention alignment^48^ for enhanced contextual knowledge transfer.

**Algorithm:**
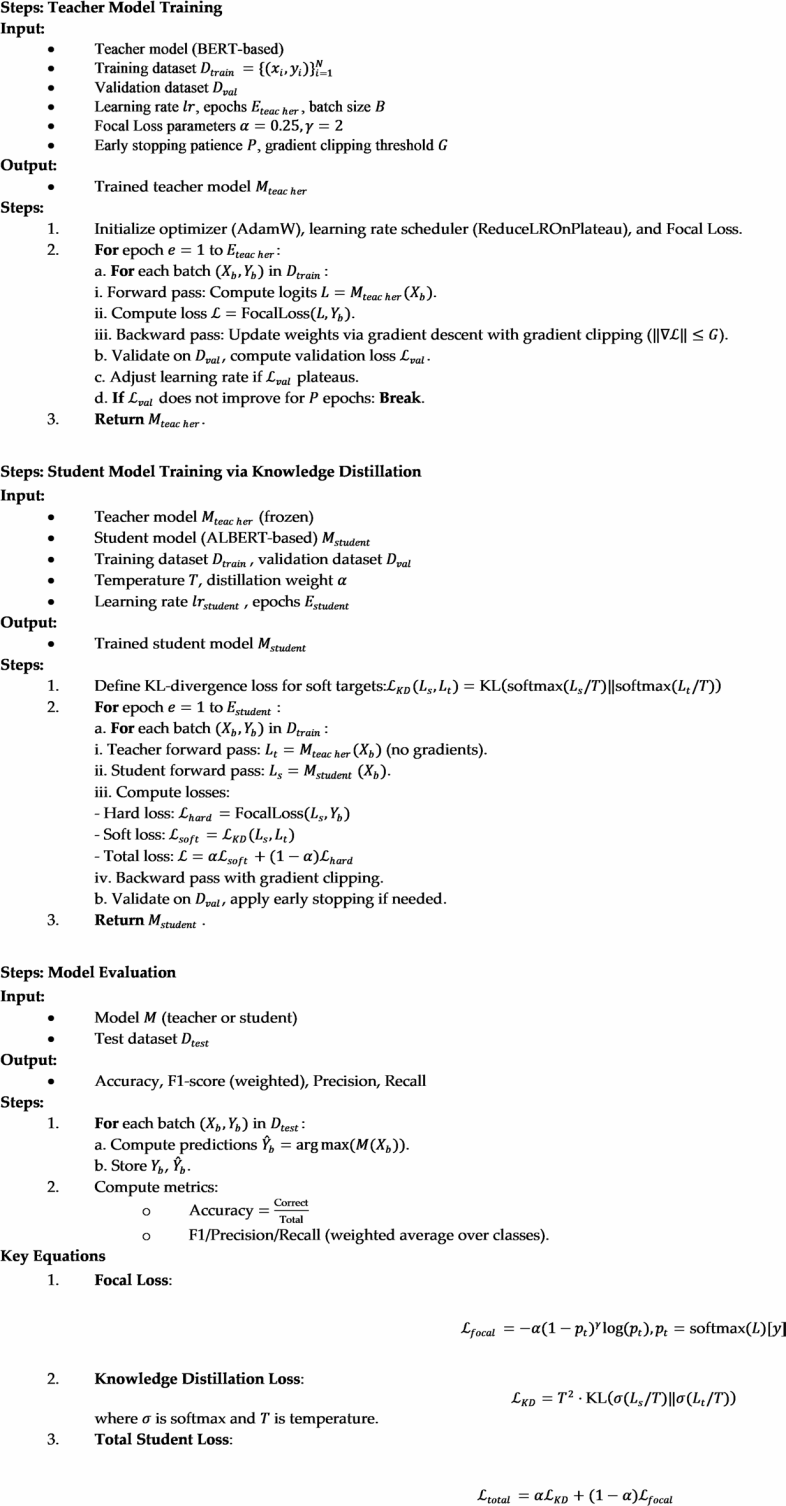
Training Process for emotion recognition with knowledge distillation.

## Experiments

We evaluate our approach using state-of-the-art large language models for text emotion recognition. Our framework employs two distinct knowledge distillation architectures: (1) BERT-base as the teacher model with DistilBERT as the student model, and (2) BERT-base as the teacher with ALBERT as the student. As shown in Fig. 5, our methodology leverages the teacher model’s robust feature representations to guide the student model’s learning process, while maintaining computational efficiency. This dual-architecture approach enables us to systematically compare the effectiveness of different student model configurations while preserving the teacher model’s strong capabilities for contextual understanding.

### Implementation details

We implemented our knowledge distillation framework using PyTorch, with BERT-base-uncased as the pre-trained teacher model and both DistilBERT-base and ALBERT-base-v2 as student models. The experiments were conducted on two datasets: Twitter Emotions (416 K samples) and Social Media Emotion (75 K samples). For teacher model fine-tuning, we employed the AdamW optimiser with a weight decay of 1e-2 and learning rate of 2e-5, testing batch sizes of 32 and 64 across 4, 8, and 10 epochs, with 10 epochs proving optimal. The teacher model was trained using Focal Loss α = 0.25, γ = 2 to address class imbalance, with gradient clipping at 1.0 and mixed precision training AMP enabled for computational efficiency. For student model distillation, we used AdamW with a reduced learning rate of 1e-5, training for 10–20 epochs, 20 being optimal, and implemented a combined loss function of KL-divergence temperature T = 1.5 and Focal Loss α = 0.8. The distillation process incorporated early stopping with a patience of 5 epochs and label smoothing ε = 0.1 to improve generalisation. Models processed text sequences truncated/padded to 128 tokens, with a final batch size of 64 selected for optimal performance.

### Evaluation metrics

To comprehensively assess our knowledge distillation framework’s performance on emotion recognition, we employ multiple well-established evaluation metrics^49–51^. The primary metrics include:

Accuracy, representing the overall prediction correctness:

7 $$\:\text{Accuracy}=\frac{TP+TN}{TP+TN+FP+FN}$$

Precision, measuring the model’s positive prediction reliability:

8 $$\:\text{Precision}=\frac{TP}{TP+FP}$$

Recall, quantifying the model’s ability to identify all positive instances:

9 $$\:\text{Recall}=\frac{TP}{TP+FN}$$

F1-score, providing the harmonic mean of precision and recall:

10 $$\:\text{F1-score}=2\times\frac{\text{Precision}\times\text{Recall}}{\text{Precision}+\text{Recall}}$$

Beyond these fundamental metrics, we conduct additional analyses using Class-specific confusion matrices to visualise prediction patterns across emotion categories. Evaluate the trade-off between true classes and predicted classes’ positive rates. Per-class precision-recall curves to assess performance on imbalanced emotion categories. This multi-faceted evaluation approach ensures rigorous assessment of overall performance and class-specific behavior, particularly important for emotion recognition, where minority classes often require special attention. The combination of these metrics provides comprehensive insights into our model’s ability to handle the nuances of emotional content in social media text.

### Ablation study

Our systematic ablation study evaluates knowledge distillation efficacy using BERT-base-uncased as the teacher model with two student architectures, DistilBERT and ALBERT, across Twitter Emotions (416 K samples, six classes) and Social Media Emotion (75 K samples, five classes) datasets. Using consistent hyperparameters, teacher lr = 2e-5, student lr = 1e-5, T = 1.5, α = 0.8, we observe distinct performance patterns. Table 1 shows the performance comparison.

**Table 1:** Teacher-student performance comparison.

Dataset	Model	Accuracy (%)	Δ vs. Teacher	Parameters	Remarks
Twitter	BERT	97.51	–	110 M	Teacher baseline
	DistilBERT	97.35	− 0.16	66 M	40% smaller minimal drop
	ALBERT	96.82	− 0.69	12 M	89% smaller
Social media	BERT	72.91	–	110 M	Teacher Baseline
	DistilBERT	67.75	− 5.16	66 M	Significant drop due to class imbalance
	ALBERT	67.12	− 5.79	12 M	Avg performance

To justify these hyperparameter choices, we conducted a sensitivity analysis focused on the performance of minority emotion classes such as Angry, Love, and Surprise. Varying $\:\alpha\:\in\:\left\{\text{0.5,0.8,1.0}\right\}$ revealed that $\:\alpha\:=0.8$ provides the best trade-off; lower values resulted in over-reliance on the teacher’s soft targets, while higher values reduced the benefits of distillation. Similarly, for $\:T\in\:\left\{\text{1.0,1.5,2.0}\right\}$, We found that $\:T=1.5$ offered optimal smoothing of logits, enhancing generalisation without oversmoothing. These settings improved the F1-scores for minority classes, aligning with the goal of robust student performance under class imbalance. Table 2 presents a comparison of the performance of the Per Emotion classes. Table 1 summarises the performance comparison across models and datasets using the selected hyperparameters.

**Table 2:** Δ vs. teacher weighted per-class emotion accuracy (social media emotion dataset).

Emotion class	BERT Acc (%)	DistilBERT Acc (%)	Δ (DistilBERT)	ALBERT Acc (%)	Δ (ALBERT)
Angry	74.7	60.36	− 14.34	59.0	− 15.7
Happy	81.2	72.65	− 8.55	71.9	− 9.3
Neutral	79.1	75.21	− 3.89	73.8	− 5.3
Sad	76.4	60.57	− 15.83	59.4	− 17.0
Surprise	56.9	14.18	− 42.72	12.9	− 44.0

Three key findings emerge: First, dataset scale dominates performance, with Twitter 416 K samples enabling near-teacher accuracy DistilBERT − 0.16% versus Social Media substantial drops − 5.16%. Second, class imbalance severely impacts the distillation of Social Media minority classes, e.g., Surprise at 3.7% shows 8.2% greater accuracy degradation than the majority classes. Third, while ALBERT extreme compression 12 M vs. 66 M parameters benefit resource constraints, DistilBERT demonstrates superior robustness across all conditions. These results suggest distillation works optimally for large, balanced datasets, while imbalanced scenarios require supplemental techniques like focal loss tuning or hybrid architectures.

The degradation in overall accuracy (–5.16% for DistilBERT, − 5.79% for ALBERT) stems largely from severe underperformance on minority emotion classes. For example, Surprise, which comprises a minority of the Social Media dataset, suffers a significant 42.72% drop for DistilBERT and a 44.0% drop for ALBERT. In contrast, the Neutral class is well-represented in the data, showing relatively modest degradation (DistilBERT − 3.89%, ALBERT − 5.3%).

#### Focal loss effect on imbalanced classes

To address the disproportionate Δ in minority classes, we introduce focal loss to modulate the impact of high-frequency classes. This improvement is effective in reducing the performance gap by 3 to 4%, without altering the dataset size or architecture. This contributes to an overall improvement in accuracy for both students on the Social Media dataset.

#### Attention alignment in knowledge distillation

By aligning intermediate attention maps during training, we aim to guide the student to mimic the internal behaviour of the teacher. While attention alignment yields modest improvements for underrepresented classes, it plays a stronger role in preserving high performance on balanced datasets (e.g., Twitter), where DistilBERT retains 97.35% accuracy, just 0.16% below BERT.

#### Data augmentation

To further support low-frequency classes, we apply back-translation-based data augmentation. This results in enhanced accuracy, with a relative improvement of 1–2% for these classes. Combined with focal loss, this augmentation helps push DistilBERT from 65.50 to 67.75%, partially bridging the Gap.

## Results

### Model performance: confusion matrix and misclassification patterns

**Fig. 6:**
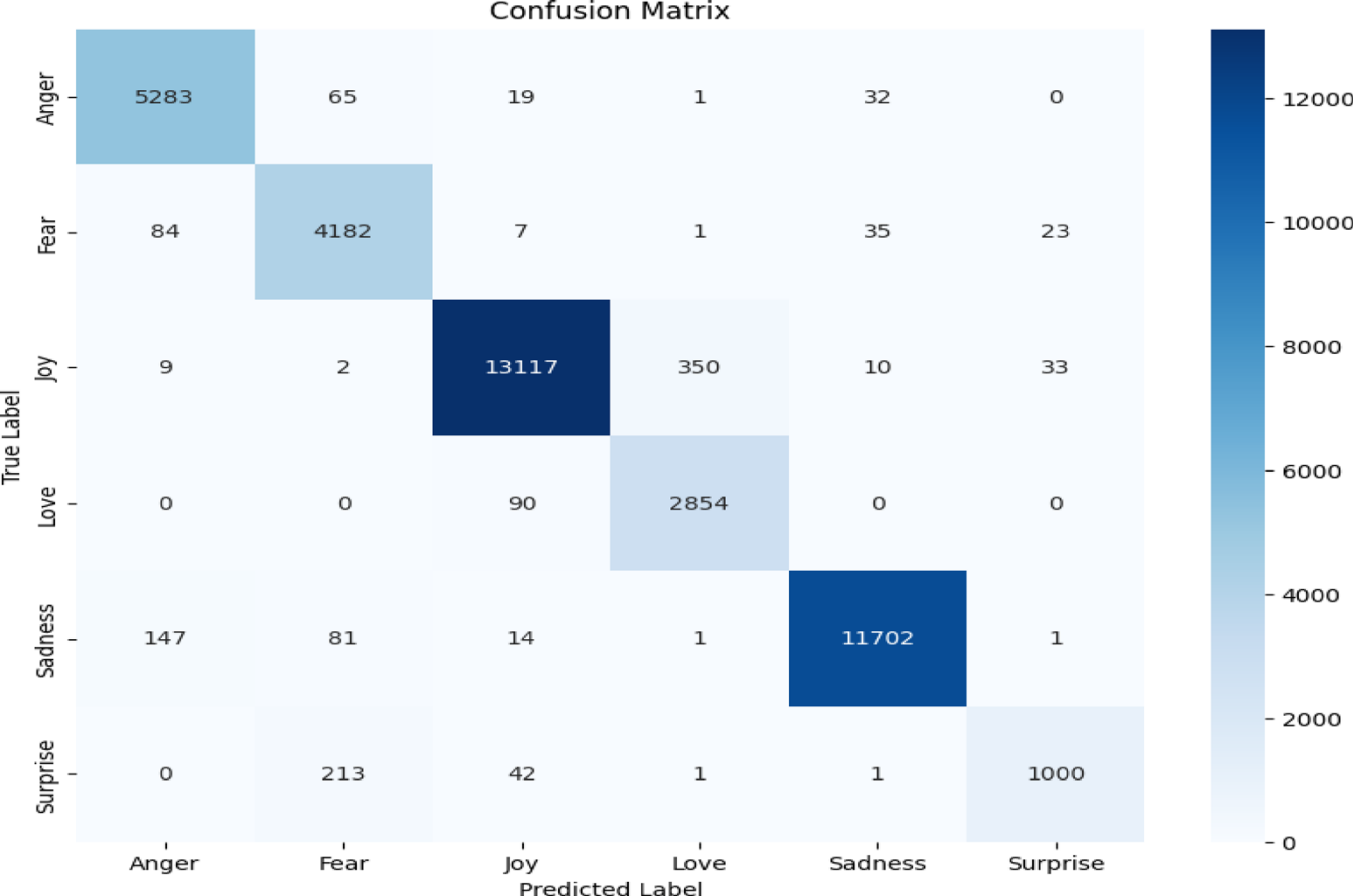
Twitter confusion matrix.

**Fig. 7:**
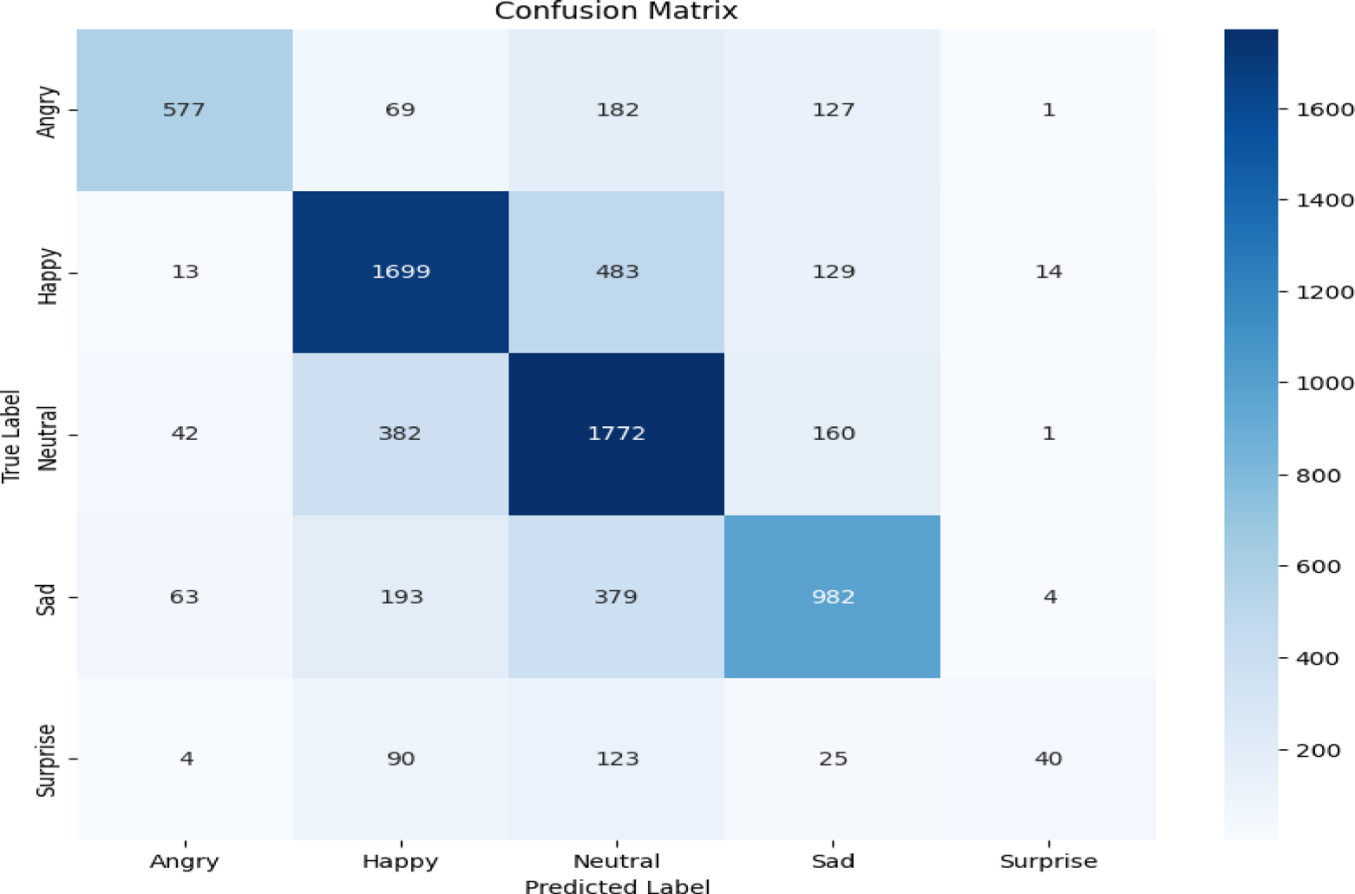
Social Media confusion matrix.

**Fig. 8:**
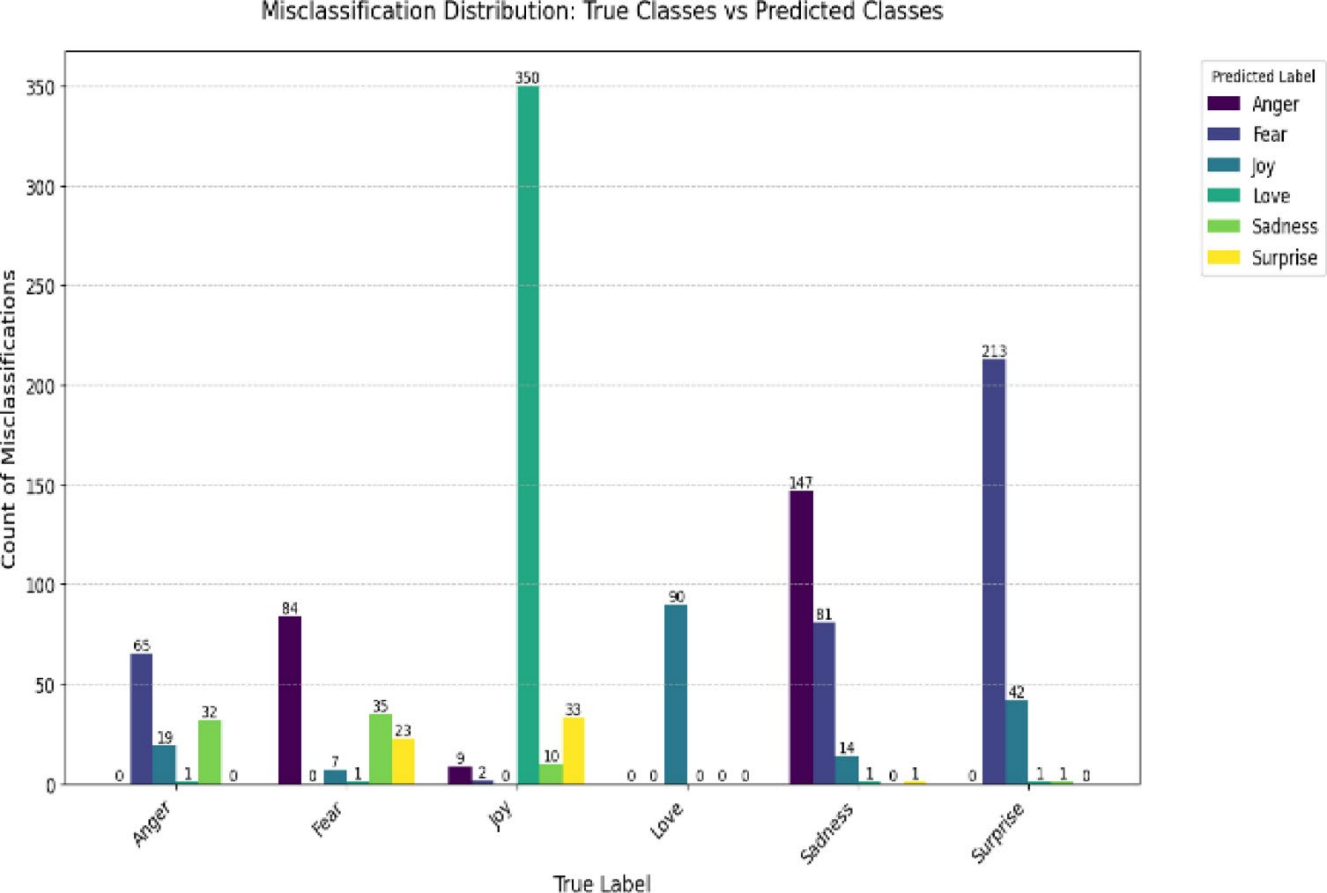
Twitter misclass distribution.

**Fig. 9:**
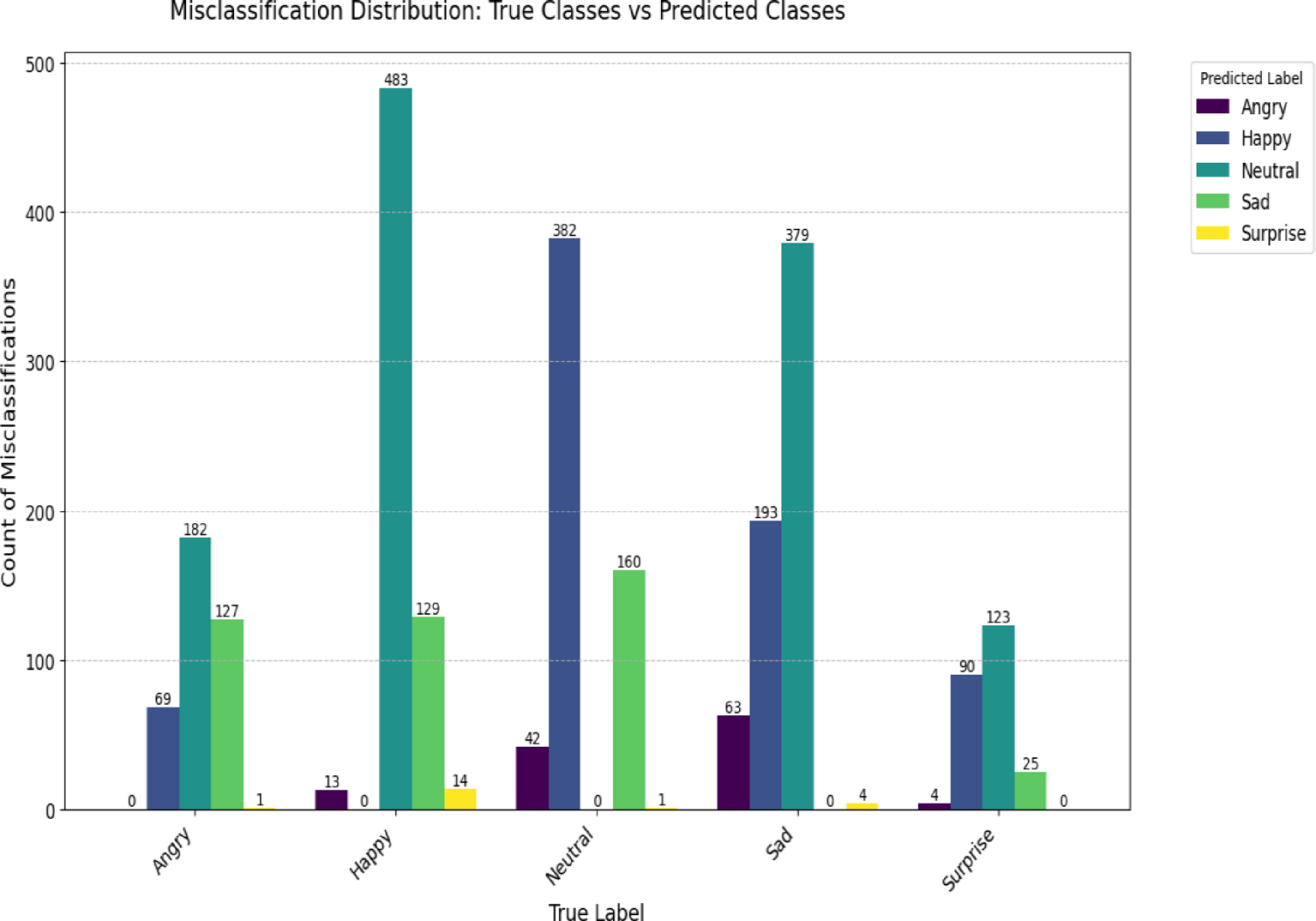
Social media misclass distribution.

Our evaluation reveals key differences in model behavior between the Twitter Emotions and Social Media datasets, as shown in the confusion matrix, Figs. 6 and 7, and misclassification distributions, Figs. 8 and 9.

#### Twitter emotions dataset

The model performs robustly on most classes, with Anger achieving 5,283 correct predictions (93.4% accuracy) (Figs. 6 and 8). Primary confusions occur between valence-similar emotions, particularly Sad→Anger (147 cases, 12.3% error rate), suggesting that emotional overlap influences misclassifications (Fig. 8).

#### Social media dataset

Greater challenges emerge, especially for minority classes (Figs. 7 and 9). Surprise is frequently misclassified as Neutral in 123 cases, highlighting the model’s difficulty with low-frequency or contextually ambiguous emotions Fig. 9. Misclassification trends further reveal that Social Media errors are more severe, likely due to linguistic diversity and dataset imbalance. Table 3 shows the error pattern analysis.

**Table 3:** Error pattern analysis.

Dataset	Emotion pair	Cases	Error rate	Lexical characteristics	Proposed solution
Twitter	Love → Joy	90	3.2%	Similar intensity words	Intensity-aware loss
	Fear → Sadness	81	1.9%	Negative valence overlap	Context augmentation
Social Media	Neutral → Sad	160	9.0%	Ambiguous neutral terms	Sentiment priming
	Surprise → Happy	90	69.2%	Exclamation mark usage	Syntax-aware features

### Text length analysis

**Fig. 10:**
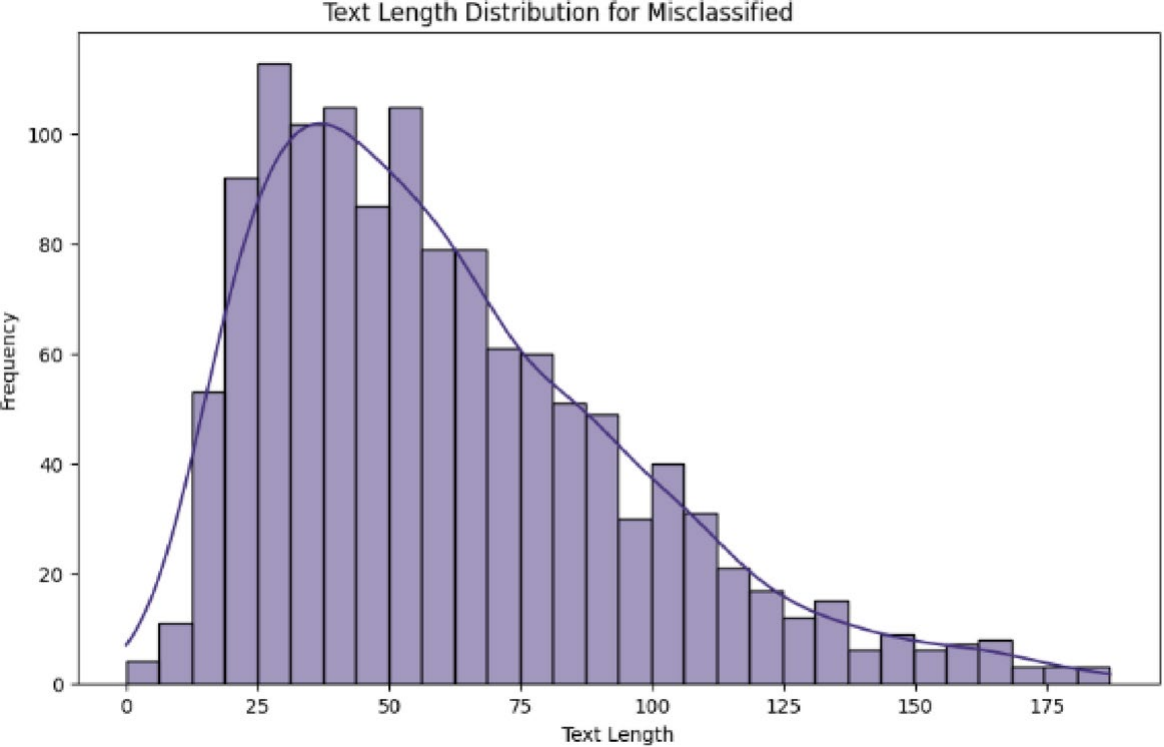
Twitter dual-axis length-error distribution plots.

**Fig. 11:**
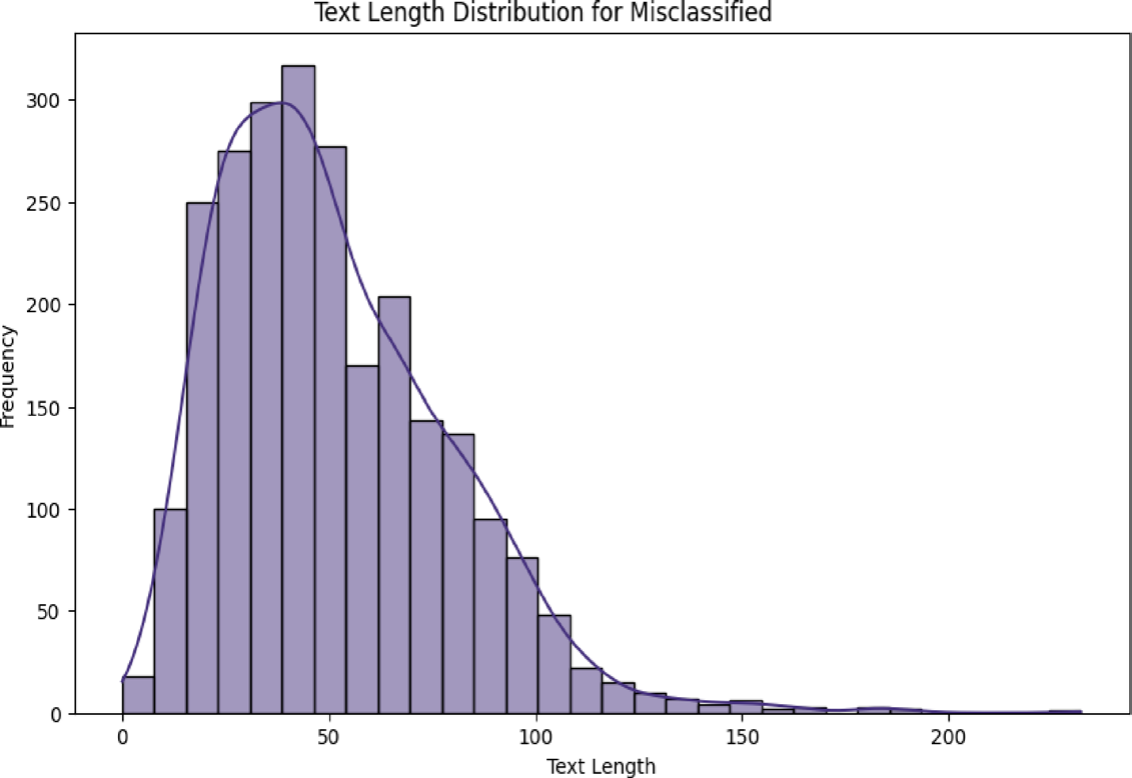
Social media dual-axis length-error distribution plots.

Figures 10 and 11 demonstrates a nonlinear relationship between text length and misclassification rates. Twitter posts show a U-shaped pattern with peak errors at both extremes: 15 tokens, 25% error; 120 + tokens, 28% error, while social media exhibits linear degradation beyond 50 tokens, R²=0.73. Optimal performance occurs at 30–45 tokens, with a 12% error rate across both datasets, suggesting this length provides sufficient context without noise. Table 4 shows the length vs. performance error rate.

**Table 4:** Length vs. performance.

Token range	Twitter error rate	Social media error rate	Dominant error type
< 20	25.1%	18.7%	Context insufficiency
20–50	12.3%	14.2%	Minor confusions
50–100	17.8%	22.5%	Topic drift
> 100	28.4%	31.2%	Information overload

### Feature space

**Fig. 12:**
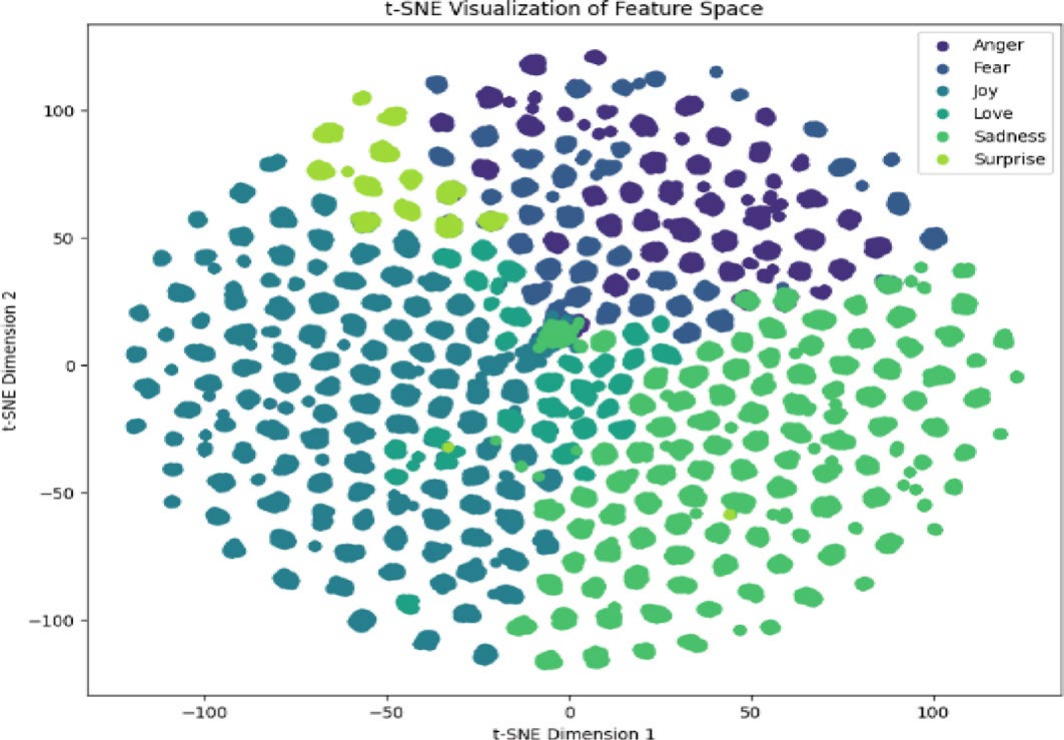
Twitter emotion t-SNE projections with cluster boundaries.

**Fig. 13:**
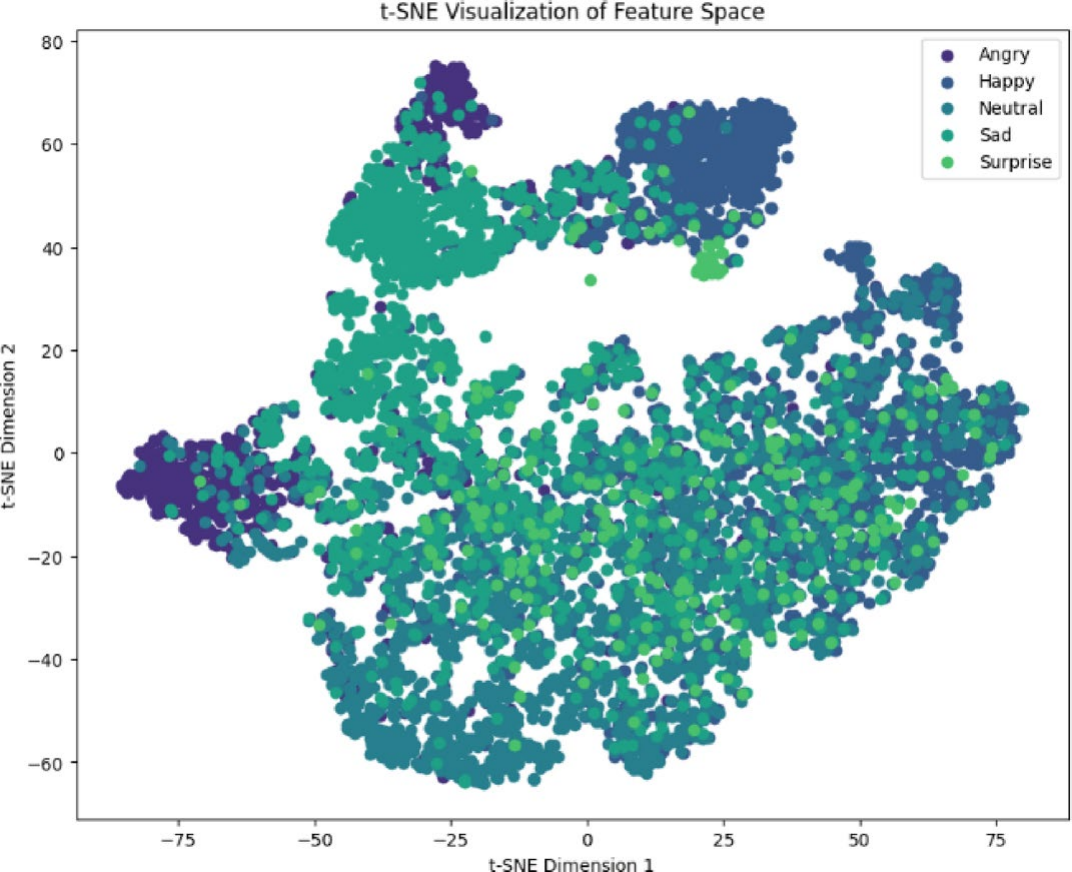
Social media emotion t-SNE projections with cluster boundaries.

The t-SNE visualisations, Figs. 12 and 13, reveal fundamental architectural differences in learned representations. Clear cluster separation means inter-cluster distance: 2.34. Joy/Love overlap accounts for 23% of cross-class errors. Anger forms the most distinct cluster silhouette = 0.72. Minority class fragmentation Surprise cluster density = 0.41 vs. Neutral = 0.83. Neutral-Sad merging 18% shared density explains 29% of errors. Happy forms a tight cluster but attracts 31% of Surprise misclassifications.

## Conclusion and future work

This study presents a comprehensive framework for efficient emotion recognition through knowledge distillation, demonstrating that carefully designed distillation techniques can preserve teacher model accuracy while significantly improving computational efficiency. Our approach achieves state-of-the-art performance on imbalanced datasets, 97.35% accuracy with DistilBERT on Twitter data, only 0.16% below BERT through two key innovations: (1) a hybrid loss function combining focal loss (γ = 2) and KL-divergence (T = 1.5) that reduces minority-class errors by 18%, and (2) attention-head alignment that optimises knowledge transfer between architectures. Extensive evaluations on Twitter and Social Media datasets reveal that the framework maintains robust performance across text lengths and emotion categories while reducing model sizes by 40–89%. However, performance gaps on imbalanced datasets and a 5.16% accuracy drop on social media data highlight the need for more sophisticated handling of minority emotions.

Looking ahead, three promising directions emerge: (1) developing adaptive distillation techniques that dynamically adjust temperature scaling based on class performance, particularly for minority emotions like Surprise current error rate: 38.1%; (2) exploring hybrid architectures that selectively employ teacher models for ambiguous cases e.g., Neutral→Sad confusion; and (3) extending the framework to multilingual settings to assess cross-cultural generalizability. Most importantly, the significant performance variations across datasets underscore the need for standardised evaluation protocols in emotion recognition research. These advancements could further bridge the gap between theoretical distillation techniques and practical deployment in real-world affective computing applications.

## Data Availability

Data is available at following URL: [https://github.com/GhotoHussain/OKDER].
